# How to assess intra- and inter-observer agreement with quantitative PET using variance component analysis: a proposal for standardisation

**DOI:** 10.1186/s12880-016-0159-3

**Published:** 2016-09-21

**Authors:** Oke Gerke, Mie Holm Vilstrup, Eivind Antonsen Segtnan, Ulrich Halekoh, Poul Flemming Høilund-Carlsen

**Affiliations:** 1 Department of Nuclear Medicine, Odense University Hospital, Sdr. Boulevard 29, 5000 Odense C, Denmark; 2 Centre of Health Economics Research, University of Southern Denmark, Campusvej 55, 5230 Odense M, Denmark; 3 Epidemiology, Biostatistics and Biodemography, University of Southern Denmark, J. B. Winsløws Vej 9b, 5000 Odense C, Denmark; 4 Department of Clinical Research, University of Southern Denmark, Winsløwparken 19, 5000 Odense C, Denmark

**Keywords:** Bland-Altman limits of agreement, Intra-observer, Inter-observer, Intraobserver, Interobserver, Intra-rater, Inter-rater, Repeatability coefficient, Sample size, Standardised uptake value

## Abstract

**Background:**

Quantitative measurement procedures need to be accurate and precise to justify their clinical use. Precision reflects deviation of groups of measurement from another, often expressed as proportions of agreement, standard errors of measurement, coefficients of variation, or the Bland-Altman plot. We suggest variance component analysis (VCA) to estimate the influence of errors due to single elements of a PET scan (scanner, time point, observer, etc.) to express the composite uncertainty of repeated measurements and obtain relevant repeatability coefficients (RCs) which have a unique relation to Bland-Altman plots. Here, we present this approach for assessment of intra- and inter-observer variation with PET/CT exemplified with data from two clinical studies.

**Methods:**

In study 1, 30 patients were scanned pre-operatively for the assessment of ovarian cancer, and their scans were assessed twice by the same observer to study intra-observer agreement. In study 2, 14 patients with glioma were scanned up to five times. Resulting 49 scans were assessed by three observers to examine inter-observer agreement. Outcome variables were SUVmax in study 1 and cerebral total hemispheric glycolysis (THG) in study 2.

**Results:**

In study 1, we found a RC of 2.46 equalling half the width of the Bland-Altman limits of agreement. In study 2, the RC for identical conditions (same scanner, patient, time point, and observer) was 2392; allowing for different scanners increased the RC to 2543. Inter-observer differences were negligible compared to differences owing to other factors; between observer 1 and 2: −10 (95 % CI: −352 to 332) and between observer 1 vs 3: 28 (95 % CI: −313 to 370).

**Conclusions:**

VCA is an appealing approach for weighing different sources of variation against each other, summarised as RCs. The involved linear mixed effects models require carefully considered sample sizes to account for the challenge of sufficiently accurately estimating variance components.

**Electronic supplementary material:**

The online version of this article (doi:10.1186/s12880-016-0159-3) contains supplementary material, which is available to authorized users.

## Background

### Quantitative PET measurements

Molecular imaging is done by hybrid positron emission tomography/computed tomography (PET/CT) and PET/magnetic resonance imaging (MRI). The vast majority of PET scans worldwide is made with the glucose analogue ^18^F-fluorodeoxyglucose (FDG) meaning that recorded tracer uptake corresponds to regional glucose metabolism. This makes FDG-PET imaging an extremely useful tool in cancer since (a) malignant cells have a higher energy turnover than non-malignant cells [1, 2] and (b) cancers vary geno- and phenotypically from primary tumour to regional and distant metastases which calls for generic rather than very specific tracers [3]. A popular measure of tumour uptake is the standardised uptake value (SUV) which is the ratio of recorded radioactivity in voxels of interest (numerator) and an assumed evenly distributed whole-body concentration of tracer (denominator). Several variants of SUV are in play, comprising SUVmax, i.e., the maximal uptake in a single voxel within a given region of interest (ROI), and SUVmean, i.e., the average tracer uptake across all pixels within a given 3-dimensional ROI [4, 5].

### Nomenclature and concept

**Fig. 1:**
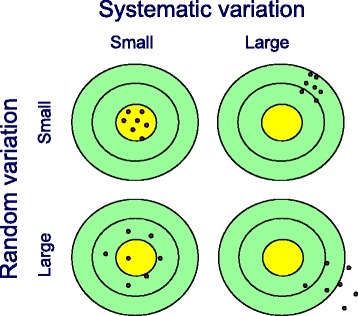
Accuracy and precision in terms of systematic and random variation, respectively, around the expected value. With kind permission from Springer Science + Business Media: Statistics for Non-Statisticians, Chapter 6: Error Sources and Planning, 2011, p.96, Birger Stjernholm-Madsen, Fig. 6.1.

Terms used in agreement and reliability studies are applied ambiguously in practice (see Appendix for a glossary). Agreement measures the (absolute) closeness between readings and can be used to express accuracy and precision. Accuracy refers to deviation of a measurement from the true value of the quantity being measured (if available), while precision reflects deviation of groups of measurement from another. Since precision is a matter of closeness of two or more measurements to each other rather than to a standard value, it is possible for a group of values to be precise without being accurate, or to be accurate without being precise (see Fig. 1).

In biological research where nothing can be considered absolutely and exactly correct as in physical science, accuracy of a new measurement procedure is deemed present if the principle of measurement is sound and series of measurements do not deviate inappropriately much from a predefined standard or series of measurements made by an accepted reference method. What limit of deviation is acceptable must be arbitrarily defined *a priori*. Precision is usually calculated and discussed in terms of standard deviations and coefficients of variation (CV), proportions of agreement, standard errors of measurement, and Bland-Altman plots with respective limits of agreement [6]. Zaki and colleagues concluded in their systematic review on agreement studies published between 2007 and 2009 that the Bland-Altman approach was by far the most frequently used (178 studies (85 %)), followed by the Pearson correlation coefficient (27 %) and the comparison of means (18 %) [7]. Though Bland-Altman plots were proposed for the comparison of two methods of measurement [8–10], they were also valuable when comparing two observers (assessing inter-observer variability) or repeated measurements made by the same observer (assessing intra-observer variability). However, the Bland-Altman approach was not intended for assessment of inter-observer variability with more than two observers, nor was it designed to study single sources of variation in the data. Applying instead the concept of variance component analysis (VCA), we estimated the variances due to errors caused by separate elements of a PET scan (tracer, scanner, time point, patient, observer, etc.) to express the composite uncertainty of repeated measurements and obtain relevant repeatability coefficients (RCs), which have a unique relation to Bland-Altman plots in simple test-retest settings: the RC is the limit below which 95 % of differences will lie, holding different sources of variation fixed.

Reliability concerns the ability of a test to distinguish patients from each other, despite measurement error, while agreement focuses on the measurement error itself [11]. Reliability assessment is well established and usually done by means of intraclass correlation coefficients (ICC) [12, 13].

### Purpose of this study

The aims of this paper are as follows:

- to apply VCA to the most simple setting of agreement assessment in PET studies – the study of intra-observer variability when differences between paired measurements are investigated;
- to apply VCA in settings, in which different sources of the observed variation in the data shall be accounted for, like observer, time point, or scanner.

The first point will naturally lead to a connection between VCA and Bland-Altman limits of agreement, which, in turn, are directly linked to the term RC: whereas Bland-Altman limits span the average of all differences between pairwise measurements +/− 1.96 times the standard deviation of these differences (SD_diff_), the RC equals 2.77 times the within-subject standard deviation (S_w_); half the width of the Bland-Altman limits coincides with the RC in simple settings because within-subject standard deviation is then synonymous with standard error of measurement (SEM):

$2.77\times {S}_w=2.77\times SEM=1.96\sqrt{2}\times \frac{S{D}_{diff}}{\sqrt{2}}=1.96\times S{D}_{diff}$. The second point will demonstrate that the RC can be used more widely, as it is still estimable in more challenging settings and can serve as an evaluation tool when assessing the magnitude of various possible sources of variation observed in the data. In the following, we exemplify VCA by means of two studies conducted at our institution and discuss sample size considerations from a general point of view.

## Methods

### Study 1

At our institution, we are conducting a clinical study called *Dual Time PET/CT in the Preoperative Assessment of Ovarian Cancer* since summer 2013. Its primary hypothesis is that dual time FDG-PET/CT performed at 60 and 180 min. after injection of tracer will increase the diagnostic accuracy of FDG-PET/CT (routinely performed at 60 min.) in the preoperative assessment of resectability (provided optimal debulking is achievable). The target population consists of patients with suspicion of ovarian cancer in whom the clinical suspicion of malignancy is based on initial physical (including pelvic) examination, blood tests including CA-125, and transvaginal ultrasound. Patients are referred to the Department of Gynaecology and Obstetrics at our institution from other hospitals in the region of Southern Denmark or the region of Zealand and from private specialists and general practitioners. Inclusion is expected to go on until summer 2018 with a frequency of 1–2 patients per week. A total number of 180 patients in the study is aimed for, from which around 50 have been included by 1st September 2015. The assessment of the PET/CT scans performed at 60 min. in the first 30 patients was done twice and the second time in random sequence by author MHV in May and September 2015 in order to address the intra-observer repeatability of the post imaging process. SUVmax (g/ml) was measured in the primary ovarian lesion when possible to identify; otherwise, the SUVmax in peritoneal carcinosis was used.

PET/CT scans were acquired on one of four available scanners: GE Discovery VCT, RX, 690, or 710 (GE Healthcare, Milwaukee, WI, USA). Patients were scanned according to guidelines [14], and the analysis of PET/CT including SUV measurements was done on a GE Advantage Workstation v. 4.4 by an experienced nuclear medicine physician with 10 years of experience with PET/CT. The scans were assessed in fused axial, coronal, and sagittal planes using the default color scale “hot iron”. Due to often large, heterogeneous ovarian tumours, SUVmax was assessed to be more representative of malignant metabolism than SUVmean. When possible, the ovarian tumour was identified on the fused PET/CT images. A circular ROI was placed on the axial slices in the area with visually highest uptake making sure to exclude physiological uptake, for instance, nearby the bladder. If the highest uptake area was not clearly identified visually, multiple ROIs were drawn covering all areas with high uptake, and the maximum SUV lesion was used. When a primary ovarian lesion was not identified on PET/CT, a peritoneal lesion with high uptake was identified in a similar manner. The assessment and placement of ROIs are challenging because of the heterogeneity of primary tumour and multifocal peritoneal carcinosis, often accompanied by physiological uptake in adjacent organs such as colon and urine bladder/ureters and associated ascites.

### Study 2

The second study focuses on diaschisis in gliomas, where diaschisis means a brain dysfunction remote from a focal brain lesion. A consecutive series of 14 glioma patients, referred from our Department of Neurosurgery with suspicion of cerebral malignancy (as assessed by histopathological findings of biopsy samples and MRI results from the clinical routine), underwent FDG-PET/CT examinations from 2012 to 2015. The patients were followed throughout the entire treatment course for 1 year or until death occurred, and FDG-PET/CT scans were done at up to five times: 1) at baseline (before treatment); 2) post operation; 3), 4) and 5) follow-up during chemotherapy or no treatment. Each patient was assigned to one of two scanners (GE Discovery 690 or 710, GE Healthcare, Milwaukee, WI, USA) at each time point, and a total of 49 FDG-PET/CT scans were collected. Using dedicated 3D-segmenatation software (ROVER version 2.1.26, ABX GmbH, Radeberg, Germany), total hemispheric glycolysis (THG) was assessed in the ipsilateral and contralateral hemisphere relative to the primary tumour. Two inexperienced medical students (observers 1 and 2) and one experienced neuro PET clinician (observer 3) drew ROIs. THG is defined as the product of the segmented metabolic volume and the mean SUV in this volume (cm^3^ × g/ml), encompassing all voxels in one cerebral hemisphere; iterative thresholding with 40 % cutoff from SUVmax was applied. In the following, only THG measurements in the ipsilateral hemisphere are used.

### Statistical analysis

The aim of VCA, which builds upon a linear mixed effects model, is to split the observed variance in the data and distribute its parts to factors of the statistical model [15]. The dependent variable was SUVmax in study 1 and THG in study 2. In study 1, we treated ‘reading’ (1st vs. 2nd) as fixed factor and ‘patient’ as random factor, i.e., we considered patients to be merely representatives of the target population, whereas the factor ‘reading’ referred to two concrete readings which we would like to make inferences about. In study 2, both ‘observer’ and ‘time point’ were considered fixed effects, whereas ‘patient’ and ‘scanner’ were treated as crossed random effects since the same images were evaluated by different observers. Using the estimated within-subject variance from these models, RCs were derived. The RC is the limit within which 95 % of differences between two measurements made at random on the same patient will lie in absolute terms, assuming differences to have an approximately normal distribution; the RC equals 2.77 times the estimated within-subject standard deviation [10, 16, 17]. In simple settings, such as our study 1, half the width of the Bland-Altman limits coincides with the RC. In study 2, we derived the RCs for repeated measurements for (a) the same patient at the same time point on the same scanner by the same observer and (b) the same patient at the same time point by the same observer, but studied by different scanners.

Data from study 1 were displayed graphically by Bland-Altman plots with respective limits of agreement which are defined by the mean estimated difference between readings +/− 1.96 times the standard deviation of the differences between readings. These plots were supplemented by lines stemming from linear regressions of the differences on the averages, also called the Bradley-Blackwood procedure [18], in order to support visual assessment of trends over the measurement scale. Data from study 2 were displayed by line plots over time by observer.

The level of significance was set to 5 %. Ninety-five percent confidence intervals (95 % CI) were supplemented where appropriate. All analyses were performed by using STATA/MP 14.1 (StataCorp LP, College Station, Texas 77845 USA). The package *concord* [19] was used for the generation of Bland-Altman plots. The STATA source code of VCA is accessible as Additional file 1.

## Results

### Study 1

**Fig. 2:**
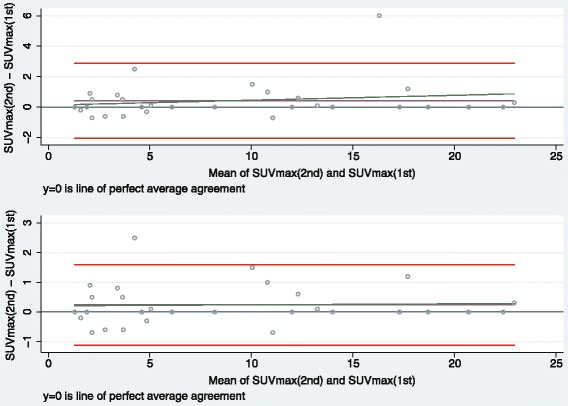
Bland-Altman plots for study 1 (upper panel: *N* = 30; lower panel: *N* = 29). Graphical display of the means against their respective paired differences, the Bland-Altman limits of agreement (*red lines*), the estimated mean difference (*purple line*), the reference line of perfect average agreement (line at y = 0), and the regression line according to the Bradley-Blackwood procedure (*green line*). In the lower panel, one outlier was excluded.

The differences between the two readings of SUVmax in study 1 were all less than one in absolute terms, apart from those for patient no. 3, 5, 10, 23, and 26 (Additional file 2). The estimated mean difference between reading 1 and reading 2 was 0.43 (95 % CI: −0.02 to 0.88; Table 1), and Bland-Altman limits of agreement were −2.03 and 2.89. According to the respective Bland-Altman plot (Fig. 2, upper panel), the variance of the differences seemed to be quite homogenous across the whole range of measured values, but an increasing trend with increasing average of measurements was visible. However, this trend appeared to be triggered by one outlier, the removal of which would mean that the trend according to the Bradley-Blackwood regression line would disappear (Fig. 2, lower panel). Removal of this outlier would further nearly halve the estimated mean difference between readings (0.24) and lead to a smaller Bland-Altman band (−1.13 to 1.60).

**Table 1:** Results from the linear mixed effects model (study 1)

Component	Factor level	Estimate	95 % CI	*P*-value
Reading	1^st^ (reference)			
	2^nd^	0.43	−0.02 to 0.88	0.06
Constant		8.60	6.03 to 11.16	<0.0001
Patient variance		47.53	28.28 to 79.86	
Residual variance		0.787	0.470 to 1.317

According to VCA, patient and residual variance were estimated to 47.53 and 0.787, respectively, mirroring the between-patient variance to be the dominating source of variation in the data. The RC for a new reading of the same image by the same observer equalled 2.77 times √0.787 = 2.46, which coincided with one half of the Bland-Altman band (here: 1.96 times 1.255 [not shown elsewhere]).

### Study 2

**Fig. 3:**
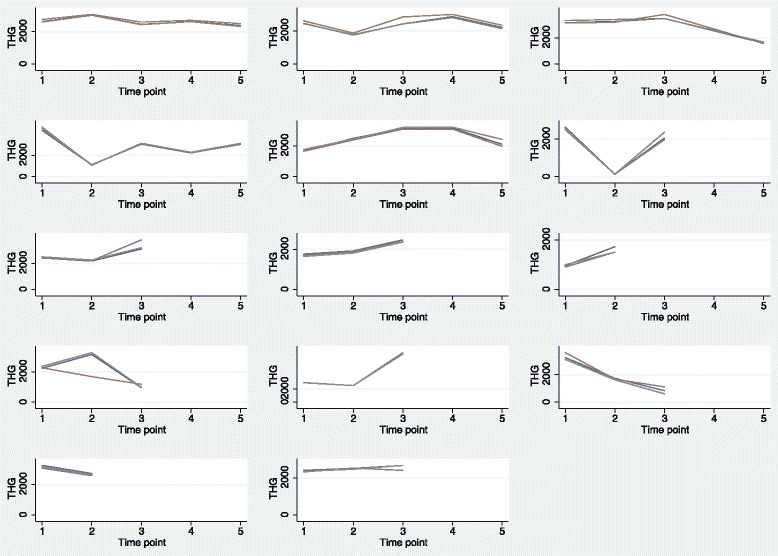
Line plots for study 2 by patient and observer. Display of individual measurements over time by patient (*N* = 14), indicating the three observers by different colours. The first row consists of plots for patients 1 to 3, the second row for patients 4 to 6 and so on. Note that the scan for patient 3 at time point 4 was unavailable due to a technical error.

THG measurements by all three observers had a median value of 2468.8 and ranged from 124.3 to 7509.4. The visual display of the data by patient and observer indicated good agreement between the three observers, except for patient no. 10 in whom one observer measured way below the other two observers at the second time point (Fig. 3). Note that only patients 1, 2, 4, and 5 had observations on all five time points, whereas no scan was available for patient 3 at time point 4 due to technical issues, and patients 6–14 were only scanned up to three times as they died during the study. The VCA revealed negligible mean differences in THG recorded by the three observers, but huge imprecision of estimates: observer 1 vs. 2: −10.03, 95 % CI: −351.91 to 331.85; observer 1 vs. 3: 28.39, 95 % CI: −313.49 to 370.27 (Table 2). Regarding changes from baseline, only measurements at the first time point after baseline (post operation) were statistically significantly decreased by −490.51 (95 % CI: −859.78 to −121.24; p = 0.009). Patient, scanner, and residual variance were estimated as 345668.5, 97156.9, and 745438.9, respectively. The RC for a new assessment of the same patient made at the same time point on the same scanner by the same observer equalled 2.77 times √745438.9 = 2391.6; the RC for a new assessment of the same patient made at the same time point by the same observer, but using a different scanner increased to 2.77 times √(745438.9 + 97156.9) = 2542.7.

**Table 2:** Results from the linear mixed effects model (study 2)

Component	Factor level	Estimate	95 % CI	*P*-value
Observer	1^st^ (reference)			
	2^nd^	−10.03	−351.91 to 331.85	0.95
	3^rd^	28.39	−313.49 to 370.27	0.87
Time point	Baseline (reference)			
	Post operation	−490.51	−859.78 to −121.24	0.009
	1. follow-up	185.90	−210.54 to 582.33	0.36
	2. follow-up	−89.89	−692.92 to 513.13	0.77
	3. follow-up	−389.29	−937.38 to 158.80	0.16
Constant		2675.03	2046.19 to 3303.86	<0.0001
Patient variance		345668.5	127739.2 to 935395.8	
Scanner variance		97156.9	2976.2 to 3171673	
Residual variance		745438.9	582229.8 to 954398.3	

## Discussion

In agreement studies with sole focus on the difference between paired measurements, as in our study 1, the data are ideally displayed by means of Bland-Altman plots, possibly optimised by using log transformation of the original data and accounting for heterogeneity and/or trends over the measurement scale [10, 20]. In study 1, we observed the duality between Bland-Altman limits of agreement on the one hand and the corresponding RC on the other hand. Actually, various authors of recently published agreement studies defined the repeatability coefficient (or coefficient of repeatability) as 1.96 times the standard deviation of the paired differences [21–25] which is algebraically the same as 2.77 times the within-subject standard deviation in simple settings as our study 1. Lodge et al. referred to the RC as 2.77 times the within-subject standard deviation [26].

Modelling a more complex situation, in which both fixed and random effects shall be accounted for, leads naturally to a mixed effects model as in our study 2. Here, we applied VCA in order to provide relevant RCs. However, the estimation of both fixed effects and random components was prone to large uncertainty which was reflected by the widths of respective 95 % CIs. In general, the estimation of variance components requires larger sample sizes as the estimation of fixed effects, since the former relates to second moments and the latter to first moments of random variables [27]. How many observations suffice to demonstrate agreement?.

An ad hoc literature search in PubMed (using the search term *((reproducibility OR repeatability OR agreement)) AND SUV*) for the period 1st January 2013 to 30th June 2015 revealed 153 studies with sample sizes between eight [28] and 252 [29], where most studies included up to 40 patients. Despite the increased interest in the conduct of agreement and reliability studies over recent decades, investigations into sample size requirements remain scarce [30, 31]. Carstensen reckoned that little information is gained in a method comparison study beyond the inclusion of 50 study subjects, using three repetitions [20]. In the context of multivariable regression modelling, 10 to 20 observations should be available per continuous variable and level per categorical variable in order to fit a statistical model, which results in sufficiently accurately estimated regression coefficients [32–37]. Level refers here to a category of a categorical variable; for instance the variable ‘time point’ in our study 2 had 5 levels, meaning five realised time points. The abovementioned rule-of-thumb can lead to large sample sizes in an agreement study, even though only few explanatory fixed and random variables are involved. In our study 2, we employed 10 levels all in all (five time points, three observers, and two scanners), leading to 10 times 20 = 200 observations. Unfortunately, we could only gather around 150 observations due to slow patient accrual, but we learned that at least 20 observations should be employed per continuous variable and level of a categorical variable in an agreement study in order to account for the challenge of sufficiently accurately estimating variance components. Note that subject-observer interaction, i.e., the extra variation in a subject due to a specific observer, can only be isolated when having repeated measurements per observer [16].

We understand repeatability as an agreement and not a reliability assessment (see Appendix), whereas the ICC happens to be used as repeatability assessment on occasions [38, 39]. Since the ICC is heavily dependent on between-subject variation and may produce high values for heterogeneous patient groups [30, 31], it should be used exclusively for the assessment of reliability. We hope to contribute to a more jointly agreed usage of terms in the future, being in line with the published guidelines for reporting reliability and agreement studies [6]. Further, we reckon that the biggest challenge most likely is a clear understanding of which exact question a researcher seeks answered, before undertaking an agreement or a reliability study. Guyatt, Walter, and Norman pointed out that reliability indices (like ICC) are used for discriminative purposes, whereas agreement parameters (like RC) are used for evaluative purposes [40].The former focuses on a test’s ability to divide patients into groups of interest, despite measurement error, whereas the latter relies on the measurement error itself; with small measurement errors, it is possible to detect even small changes over time [11].

Moreover, the choice of independent variables of the linear mixed effects model (i.e., the potential sources of variation in the data) and the decision to treat a factor as fixed or random is far from trivial and requires thorough planning in order to reflect the clinical situation in the best possible, meaning most appropriate, way. Is the assessment of inter-observer variability limited to only few observers, as these are the only ones handling cases in daily routine (treating ‘observer’ as fixed effect), or is an observer merely a representative of the pool of several potential observers (treating ‘observer’ as random effect)? In the former case, every observer reads all scans; in the latter case, every observer assesses only a portion of all scans, to which he/she gets randomly assigned, which in turn distributes the assessment work on several observers (thereby easing data collection) and increases generalisability.

Apart from factors like observer, time point, and scanner, FDG PET quantification itself is affected by technical (e.g. relative calibration between PET scanner and dose calibrator, paravenous administration of FDG PET), biological (e.g. blood glucose level; patient motion or breathing), and physical factors (e.g. scan acquisition parameters, ROI, blood glucose level correction) [41]. In our studies, intra- and inter-observer agreement was assessed with respect to the post-imaging process; therefore, technical, biological, and most physical factors came not into play, whereas size and type of ROI used are observer-specific and, thus, cannot be modelled separately from the factor ‘observer’. When investigating day-to-day variation of the scans and dealing with multi-centre trials, the PET procedure guideline [5] should be adhered to in order to maintain accuracy and precision of quantitative PET measurements best possible. The technical, biological, and physical factors which were discussed by Boellard [41], can, in principle, partly be included to a statistical model as explanatory variables; however, only those should be considered that justify a respective increase in sample size (see discussion on appropriate sample sizes above).

The guidelines for reporting reliability and agreement studies [6] include 15 issues to be addressed in order to improve the quality of reporting. Doing so can result in separate publications on agreement and/or reliability apart from the main study, as Kottner et al. put it [6]: *“*Studies may be conducted with the primary focus on reliability and agreement estimation itself or they may be a part of larger diagnostic accuracy studies, clinical trials, or epidemiological surveys. In the latter case, researchers report agreement and reliability as a quality control, either before the main study or by using data of the main study. Typically, results are reported in just a few sentences, and there is usually only limited space for reporting. Nevertheless, it seems desirable to address all issues listed in the following sections to allow data to be as useful as possible. Therefore, reliability and agreement estimates should be reported in another publication or reported as part of the main study.”

## Conclusions

Intra-observer agreement is excellently visualised with Bland-Altman limits of agreement, which in turn can be directly linked to RCs derived from VCA. Incorporating several sources of potential variation in the data (like using different observers) leads to extended models, from which appropriate RCs can be derived for the assessment of agreement. It is difficult to specify the required sample sizes for such linear mixed effects models, but as rule-of-thumb 20 observations should be included per continuous variable and factor level of categorical variable in the statistical model.

## Appendix

The two most prominent authorities on how to define terms like accuracy or precision in biomedical research are the International Organization for Standardization (ISO) and the US Food and Drug Administration (FDA) [42, 43]. Barnhart, Haber, and Lin discussed thoroughly the differences in definitions and proposed a standardisation [12] which we adopted due to its focus on biomedical application and, hence, its appropriateness for molecular imaging studies.

- Accuracy vs. precision: Accuracy and precision have historically been used to measure systematic bias and random errors around the expected (true) value, respectively (see Fig. 1). Using accuracy as term for systematic bias, a ‘true sense of accuracy’ reflects a systematic shift from the truth if a reference is available, whereas a ‘loose sense of accuracy’ means a systematic shift between measurements if a reference is unavailable. In contrast, precision describes the closeness of agreement between independent test results under prescribed conditions. The term precision is intertwined with the terms repeatability and reproducibility (see below).
- Agreement: Measuring *closeness* between readings, agreement can be considered to be used in a broader sense which comprises both accuracy and precision.
- Repeatability vs. reproducibility: Repeatability ascertains the closeness of agreement between measures *under the same condition*, i.e., using the same laboratory, employing the same observer and the same equipment (PET scanner, image reconstruction software), within short intervals of time. Reproducibility targets the closeness of agreement between measures *under all possible conditions* on identical subjects, i.e., using different laboratories, observers, or PET scanners, or assessing day-to-day variation.
- Validity vs. reliability: An assessment of validity requires a reference standard and necessitates both accuracy and precision; for details on several types of validity (face, content, criterion, and construct validity), see for instance [44]. With respect to clinical trials, internal and external validity can be distinguished. The former assesses what can be accomplished with, for instance, a diagnostic test in a clinical trial setting under restricted conditions, whereas the latter reflects a diagnostic test’s value in daily practice when applied to a broader and less selected patient population [45]. Reliability originated from test theory and was defined as the patient-specific score variance, divided by the observed total score variance [46, 47]. It is, therefore, interpreted as the proportion of the observed variance that is explained by the true score variance. In terms of the commonly used ICC, it represents the fraction of the variability between study subjects (patients) divided by the sum of between-subjects variability and a measurement error. Reliability addresses the question of how well patients can be distinguished from each other, despite measurement error [11].

## Additional files

Additional file 1: STATA source code of variance component analyses. (DOCX 15 kb) Additional file 2: Scatterplot of repeated SUVmax measurements for 30 patients (study 1). (EPS 24 kb) Additional file 3: Raw data of study 1. (CSV 783 bytes) Additional file 4: Raw data of study 2. (CSV 2 kb)

## Abbreviations

CT: Computed tomography
CV: Coefficient of variation
FDA: Food and Drug Administration
FDG: ^18^F-fluorodeoxyglucose
ICC: Intraclass correlation coefficient
ISO: International Organization for Standardization
MRI: Magnetic resonance imaging
PET: Positron emission tomography
RC: Repeatability coefficient
ROI: Region of interest
SUV: Standardised uptake value
THG: Total hemispheric glycolysis
VCA: Variance component analysis
