# Supplementary material for: How to assess intra- and inter-observer agreement with quantitative PET using variance component analysis: a proposal for standardisation
Source: BMC Med Imaging. 2016 Sep 21;16:54. doi: 10.1186/s12880-016-0159-3 (PMC5031256; doi:10.1186/s12880-016-0159-3)
Supplement: Additional file 1: — STATA source code of variance component analyses. (DOCX 15 kb) [file 12880_2016_159_MOESM1_ESM.docx]

**Online Resource 1: STATA source code of variance component analyses**

**Study 1:**

import delimited "C:\temp\data_study1_2016_09_01.csv", clear

mixed suvmax reading || _all:R.patient , reml

**Study 2:**

import delimited " C:\temp\data_study2_2016_09_01.csv", clear

mixed thg i.observer i.timepoint || _all:R.patient || _all:R.scanner , reml
